# Aqua­chloridobis(1,10-phenanthroline-κ^2^ *N*,*N*′)zinc(II) chloride *N*,*N*-dimethyl­formamide solvate

**DOI:** 10.1107/S1600536808002237

**Published:** 2008-01-25

**Authors:** Li-Li Kong, Shan Gao, Li-Hua Huo, Seik Weng Ng

**Affiliations:** a School of Chemistry and Materials Science, Heilongjiang University, Harbin 150080, People’s Republic of China; b Department of Chemistry, University of Malaya, 50603 Kuala Lumpur, Malaysia

## Abstract

The Zn atom in the title salt, [ZnCl(C_12_H_8_N_2_)_2_(H_2_O)]Cl·C_3_H_7_NO, is chelated by two phenanthroline mol­ecules and is bonded to one chloride ion and one water mol­ecule, resulting in a ZnN_4_ClO octa­hedral coordination environment with the Cl and O atoms in a *cis* conformation. The cations and anions are linked by O—H⋯Cl hydrogen bonds across a center of inversion, forming a hydrogen-bonded dimeric association. The dimethyl­formamide solvent mol­ecule is disordered over two orientations in a 0.56 (1):0.44 (1) ratio.

## Related literature

The title compound is isostructural with the cobalt and nickel analogs: see Liu, Gao, Huo & Ng (2004 ▶); Liu, Liu & Zhong (2004 ▶).
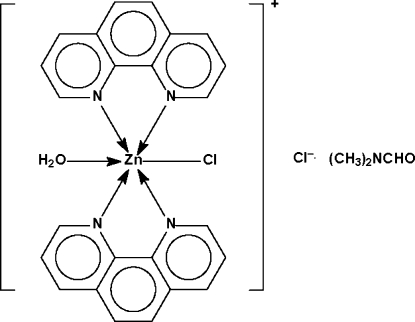


## Experimental

### 

#### Crystal data

[ZnCl(C_12_H_8_N_2_)_2_(H_2_O)]Cl·C_3_H_7_NO
*M* *_r_* = 587.79
Triclinic, 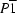

*a* = 9.6743 (3) Å
*b* = 11.6096 (5) Å
*c* = 12.7486 (5) Å
α = 67.004 (1)°
β = 85.995 (1)°
γ = 80.025 (1)°
*V* = 1298.14 (9) Å^3^
*Z* = 2
Mo *K*α radiation
μ = 1.19 mm^−1^
*T* = 295 (2) K
0.30 × 0.24 × 0.18 mm

#### Data collection

Rigaku R-AXIS RAPID diffractometer
Absorption correction: multi-scan (*ABSCOR*; Higashi, 1995 ▶) *T*_min_ = 0.660, *T*_max_ = 0.815
12819 measured reflections
5884 independent reflections
4602 reflections with *I* > 2σ(*I*)
*R*_int_ = 0.022

#### Refinement

*R*[*F* ^2^ > 2σ(*F* ^2^)] = 0.033
*wR*(*F* ^2^) = 0.103
*S* = 1.11
5884 reflections
383 parameters
61 restraints
H atoms treated by a mixture of independent and constrained refinement
Δρ_max_ = 0.43 e Å^−3^
Δρ_min_ = −0.36 e Å^−3^

### 

Data collection: *RAPID-AUTO* (Rigaku, 1998 ▶); cell refinement: *RAPID-AUTO*; data reduction: *CrystalStructure* (Rigaku/MSC, 2002 ▶); method used to solve structure: atomic coordinates taken from the iostructural Co compound (Liu *et al*., 2004 ▶ ▶); program(s) used to refine structure: *SHELXL97* (Sheldrick, 2008 ▶); molecular graphics: *X-SEED* (Barbour, 2001 ▶); software used to prepare material for publication: *publCIF* (Westrip, 2008 ▶).

## Supplementary Material

Crystal structure: contains datablocks global, I. DOI: 10.1107/S1600536808002237/hb2689sup1.cif

Structure factors: contains datablocks I. DOI: 10.1107/S1600536808002237/hb2689Isup2.hkl

Additional supplementary materials:  crystallographic information; 3D view; checkCIF report

## Figures and Tables

**Table 1:** Selected bond lengths (Å)

Zn1—N1	2.190 (2)
Zn1—N2	2.198 (2)
Zn1—N3	2.157 (2)
Zn1—N4	2.275 (2)
Zn1—O1*w*	2.090 (2)
Zn1—Cl1	2.3520 (6)

**Table 2:** Hydrogen-bond geometry (Å, °)

*D*—H⋯*A*	*D*—H	H⋯*A*	*D*⋯*A*	*D*—H⋯*A*
O1*w*—H1*w*1⋯Cl2	0.85 (3)	2.29 (3)	3.112 (2)	163 (3)
O1*w*—H1*w*2⋯Cl2^i^	0.84 (3)	2.24 (3)	3.079 (2)	172 (3)

Symmetry code: (i) 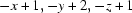.

**Fig. 1.**
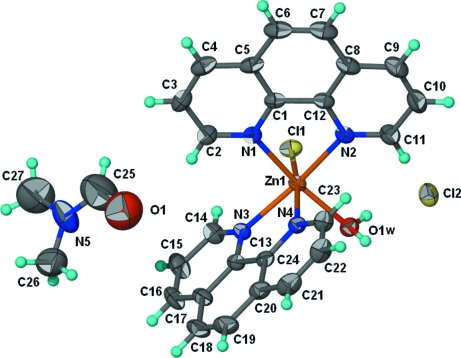
View of the molecular structure of (I); displacement ellipsoids are drawn at the 50% probability level, and H atoms as spheres of arbitrary radius. Only one disorder component of the DMF molecule is shown.

## supplementary crystallographic information

### Comment

The title compound, (I), is isostructural with its cobalt (Liu, Gao, Huo & Ng, 2004) and nickel analogs (Liu, Liu & Zhong, 2004).

The Zn atom in (I) is chelated by two phenanthroline molecules and bonded to one chloride ion and one water molecule, resulting in a ZnN_4_ClO octahedral coordination environment with the Cl and O atoms in a *cis* conformation (Table 1, Fig. 1). The cations and anions are linked by O–H···Cl hydrogen bonds across a center of inversion forming a hydrogen-bonded dimeric association. Details of the hydrogen bonds are given in Table 2.

### Experimental

Zinc diacetate dihydrate (1 mmol) and 1,10-phenanthroline (2 mmol) were dissolved in a DMF-water mixture. Several drops of hydrochloric acid were added, and the mixture set aside for the growth of crystals.. The mixture was filtered and colorless blocks of (I) were isolated after several days.

### Refinement

The carbon-bound H atoms were placed in calculated positions (C—H = 0.93–0.97 Å) and refined as riding with *U*_iso_(H) 1.2–1.5*U*_eq_(C).

The water H-atoms were located in a difference map, and were refined with a distance restraint of O–H = 0.85±0.01 Å; their *U*_iso_ values were refined.

The DMF solvate molecule is disordered but the disorder components share a nitrogen atom. The C–O distance was restrained to 1.25±0.01 Å, the *N*–C_carbonyl_ distance to 1.35±0.01 Å and the *N*–C_methyl_ distance to 1.45±0.01 Å. The molecule was restrained to be nearly flat. The occupations of the disorder components refined to 0.56 (1):0.44 (1).

### Crystal data

**Table:**

[ZnCl(C_12_H_8_N_2_)_2_(H_2_O)]Cl·C_3_H_7_NO	*Z* = 2
*M**_r_* = 587.79	*F*(000) = 604
Triclinic, *P*1	*D*_x_ = 1.504 Mg m^−^^3^
Hall symbol: -P 1	Mo *K*α radiation, λ = 0.71073 Å
*a* = 9.6743 (3) Å	Cell parameters from 10359 reflections
*b* = 11.6096 (5) Å	θ = 3.1–27.5°
*c* = 12.7486 (5) Å	µ = 1.19 mm^−^^1^
α = 67.004 (1)°	*T* = 295 K
β = 85.995 (1)°	Block, colorless
γ = 80.025 (1)°	0.30 × 0.24 × 0.18 mm
*V* = 1298.14 (9) Å^3^	

### Data collection

**Table:**

Rigaku R-AXIS RAPID diffractometer	5884 independent reflections
Radiation source: fine-focus sealed tube	4602 reflections with *I* > 2σ(*I*)
graphite	*R*_int_ = 0.022
Detector resolution: 10.000 pixels mm^-1^	θ_max_ = 27.5°, θ_min_ = 3.1°
ω scans	*h* = −12→12
Absorption correction: multi-scan (*ABSCOR*; Higashi, 1995)	*k* = −15→15
*T*_min_ = 0.660, *T*_max_ = 0.815	*l* = −15→16
12819 measured reflections	

### Refinement

**Table:**

Refinement on *F*^2^	Primary atom site location: structure-invariant direct methods
Least-squares matrix: full	Secondary atom site location: difference Fourier map
*R*[*F*^2^ > 2σ(*F*^2^)] = 0.033	Hydrogen site location: inferred from neighbouring sites
*wR*(*F*^2^) = 0.103	H atoms treated by a mixture of independent and constrained refinement
*S* = 1.11	*w* = 1/[σ^2^(*F*_o_^2^) + (0.0615*P*)^2^] where *P* = (*F*_o_^2^ + 2*F*_c_^2^)/3
5884 reflections	(Δ/σ)_max_ = 0.001
383 parameters	Δρ_max_ = 0.43 e Å^−^^3^
61 restraints	Δρ_min_ = −0.36 e Å^−^^3^

### Fractional atomic coordinates and isotropic or equivalent isotropic displacement parameters (Å^2^)

**Table:**

	*x*	*y*	*z*	*U*_iso_*/*U*_eq_	Occ. (<1)
Zn1	0.69100 (2)	0.67147 (2)	0.71925 (2)	0.02999 (10)	
Cl1	0.57695 (6)	0.68729 (6)	0.88327 (5)	0.04014 (15)	
Cl2	0.39933 (7)	1.05103 (6)	0.64297 (5)	0.04871 (17)	
O1W	0.53618 (17)	0.80418 (17)	0.61027 (14)	0.0387 (4)	
H1W1	0.498 (3)	0.861 (2)	0.634 (2)	0.059 (9)*	
H1W2	0.553 (3)	0.837 (3)	0.5398 (10)	0.055 (8)*	
N1	0.88448 (17)	0.55649 (18)	0.80695 (15)	0.0324 (4)	
N2	0.83314 (19)	0.80984 (18)	0.68954 (16)	0.0352 (4)	
N3	0.61286 (18)	0.50502 (17)	0.72752 (15)	0.0316 (4)	
N4	0.78178 (18)	0.63714 (18)	0.56257 (15)	0.0317 (4)	
N5	0.7617 (3)	0.0237 (3)	0.9642 (3)	0.0874 (10)	
C1	0.9880 (2)	0.6243 (2)	0.80178 (18)	0.0324 (5)	
C2	0.9080 (2)	0.4331 (2)	0.8658 (2)	0.0402 (5)	
H2	0.8381	0.3861	0.8686	0.048*	
C3	1.0332 (3)	0.3698 (3)	0.9242 (2)	0.0464 (6)	
H3	1.0454	0.2826	0.9651	0.056*	
C4	1.1368 (3)	0.4364 (3)	0.9207 (2)	0.0481 (6)	
H4	1.2201	0.3953	0.9601	0.058*	
C5	1.1177 (2)	0.5674 (3)	0.8576 (2)	0.0400 (6)	
C6	1.2211 (2)	0.6458 (3)	0.8478 (2)	0.0509 (7)	
H6	1.3063	0.6092	0.8855	0.061*	
C7	1.1979 (3)	0.7700 (3)	0.7860 (3)	0.0532 (7)	
H7	1.2687	0.8175	0.7786	0.064*	
C8	1.0664 (2)	0.8316 (3)	0.7308 (2)	0.0439 (6)	
C9	1.0341 (3)	0.9631 (3)	0.6682 (2)	0.0530 (7)	
H9	1.1007	1.0150	0.6596	0.064*	
C10	0.9053 (3)	1.0136 (3)	0.6203 (3)	0.0553 (7)	
H10	0.8825	1.1005	0.5799	0.066*	
C11	0.8071 (3)	0.9338 (2)	0.6322 (2)	0.0456 (6)	
H11	0.7196	0.9696	0.5984	0.055*	
C12	0.9604 (2)	0.7587 (2)	0.73849 (18)	0.0339 (5)	
C13	0.6459 (2)	0.4679 (2)	0.63922 (19)	0.0324 (5)	
C14	0.5353 (2)	0.4368 (2)	0.8115 (2)	0.0421 (6)	
H14	0.5150	0.4599	0.8737	0.050*	
C15	0.4828 (3)	0.3327 (3)	0.8111 (3)	0.0546 (7)	
H15	0.4287	0.2878	0.8716	0.065*	
C16	0.5120 (3)	0.2975 (3)	0.7202 (3)	0.0527 (7)	
H16	0.4758	0.2295	0.7175	0.063*	
C17	0.5967 (2)	0.3644 (2)	0.6314 (2)	0.0415 (6)	
C18	0.6358 (3)	0.3313 (3)	0.5352 (2)	0.0525 (7)	
H18	0.6026	0.2636	0.5291	0.063*	
C19	0.7204 (3)	0.3973 (3)	0.4532 (2)	0.0541 (7)	
H19	0.7447	0.3740	0.3916	0.065*	
C20	0.7732 (2)	0.5017 (3)	0.4589 (2)	0.0417 (5)	
C21	0.8641 (3)	0.5729 (3)	0.3765 (2)	0.0530 (7)	
H21	0.8931	0.5519	0.3142	0.064*	
C22	0.9091 (3)	0.6714 (3)	0.3882 (2)	0.0501 (7)	
H22	0.9683	0.7188	0.3341	0.060*	
C23	0.8649 (2)	0.7005 (2)	0.4829 (2)	0.0412 (5)	
H23	0.8960	0.7686	0.4899	0.049*	
C24	0.7357 (2)	0.5379 (2)	0.55131 (18)	0.0322 (5)	
O1	0.8615 (13)	0.1721 (13)	0.8303 (11)	0.145 (5)	0.560 (9)
C25	0.8320 (9)	0.1203 (9)	0.9327 (10)	0.153 (5)	0.560 (9)
H25	0.8587	0.1486	0.9860	0.184*	0.560 (9)
C26	0.7138 (10)	−0.0335 (9)	0.8953 (7)	0.105 (3)	0.560 (9)
H26A	0.7384	0.0105	0.8171	0.157*	0.560 (9)
H26B	0.6137	−0.0287	0.9020	0.157*	0.560 (9)
H26C	0.7571	−0.1207	0.9204	0.157*	0.560 (9)
C27	0.7266 (10)	−0.0410 (11)	1.0865 (6)	0.137 (4)	0.560 (9)
H27A	0.8004	−0.0406	1.1329	0.206*	0.560 (9)
H27B	0.7166	−0.1269	1.1017	0.206*	0.560 (9)
H27C	0.6401	0.0027	1.1039	0.206*	0.560 (9)
O1'	0.9009 (13)	0.1667 (11)	0.8546 (11)	0.115 (4)	0.440 (9)
C25'	0.8264 (10)	0.0799 (9)	0.8675 (9)	0.100 (4)	0.440 (9)
H25'	0.8180	0.0563	0.8067	0.120*	0.440 (9)
C26'	0.7784 (13)	0.0665 (13)	1.0524 (9)	0.135 (5)	0.440 (9)
H26D	0.8736	0.0797	1.0528	0.202*	0.440 (9)
H26E	0.7569	0.0040	1.1246	0.202*	0.440 (9)
H26F	0.7161	0.1447	1.0393	0.202*	0.440 (9)
C27'	0.6808 (12)	−0.0719 (10)	0.9728 (17)	0.168 (7)	0.440 (9)
H27D	0.7428	−0.1478	0.9772	0.253*	0.440 (9)
H27E	0.6220	−0.0425	0.9069	0.253*	0.440 (9)
H27F	0.6234	−0.0895	1.0401	0.253*	0.440 (9)

### Atomic displacement parameters (Å^2^)

**Table:**

	*U*^11^	*U*^22^	*U*^33^	*U*^12^	*U*^13^	*U*^23^
Zn1	0.02905 (14)	0.03305 (16)	0.03096 (15)	−0.00828 (10)	0.00059 (10)	−0.01436 (11)
Cl1	0.0364 (3)	0.0549 (4)	0.0340 (3)	−0.0079 (3)	0.0019 (2)	−0.0225 (3)
Cl2	0.0604 (4)	0.0398 (3)	0.0463 (4)	−0.0088 (3)	0.0078 (3)	−0.0180 (3)
O1W	0.0421 (9)	0.0380 (10)	0.0353 (9)	−0.0009 (7)	−0.0029 (7)	−0.0151 (8)
N1	0.0296 (8)	0.0378 (10)	0.0310 (9)	−0.0059 (8)	0.0007 (7)	−0.0146 (8)
N2	0.0348 (9)	0.0380 (11)	0.0381 (10)	−0.0104 (8)	0.0016 (8)	−0.0187 (9)
N3	0.0311 (9)	0.0338 (10)	0.0328 (10)	−0.0085 (8)	0.0026 (7)	−0.0149 (8)
N4	0.0330 (9)	0.0334 (10)	0.0288 (9)	−0.0045 (8)	0.0006 (7)	−0.0126 (8)
N5	0.106 (2)	0.069 (2)	0.099 (2)	−0.0437 (19)	0.034 (2)	−0.0382 (18)
C1	0.0259 (9)	0.0473 (13)	0.0299 (11)	−0.0061 (9)	0.0024 (8)	−0.0216 (10)
C2	0.0375 (11)	0.0439 (14)	0.0372 (13)	−0.0055 (10)	0.0016 (10)	−0.0140 (11)
C3	0.0460 (13)	0.0473 (15)	0.0370 (13)	0.0054 (11)	−0.0050 (10)	−0.0111 (11)
C4	0.0369 (12)	0.0707 (19)	0.0369 (13)	0.0060 (12)	−0.0041 (10)	−0.0263 (13)
C5	0.0291 (10)	0.0619 (17)	0.0347 (12)	−0.0006 (11)	−0.0019 (9)	−0.0272 (12)
C6	0.0282 (11)	0.081 (2)	0.0568 (17)	−0.0059 (12)	−0.0015 (11)	−0.0421 (16)
C7	0.0324 (12)	0.080 (2)	0.0638 (18)	−0.0224 (13)	0.0055 (12)	−0.0414 (17)
C8	0.0368 (12)	0.0597 (17)	0.0487 (14)	−0.0210 (12)	0.0099 (11)	−0.0315 (13)
C9	0.0509 (15)	0.0574 (18)	0.0607 (17)	−0.0311 (14)	0.0095 (13)	−0.0259 (14)
C10	0.0678 (18)	0.0410 (15)	0.0591 (17)	−0.0207 (14)	0.0046 (14)	−0.0170 (13)
C11	0.0455 (13)	0.0433 (14)	0.0507 (15)	−0.0128 (11)	0.0008 (11)	−0.0186 (12)
C12	0.0300 (10)	0.0463 (14)	0.0334 (11)	−0.0120 (10)	0.0046 (9)	−0.0221 (10)
C13	0.0298 (10)	0.0337 (12)	0.0373 (12)	−0.0033 (9)	−0.0034 (9)	−0.0178 (10)
C14	0.0396 (12)	0.0439 (14)	0.0473 (14)	−0.0132 (11)	0.0116 (11)	−0.0217 (12)
C15	0.0525 (15)	0.0464 (16)	0.0701 (19)	−0.0239 (13)	0.0183 (14)	−0.0245 (14)
C16	0.0493 (14)	0.0397 (14)	0.078 (2)	−0.0167 (12)	0.0029 (14)	−0.0287 (14)
C17	0.0383 (12)	0.0397 (13)	0.0556 (15)	−0.0055 (10)	−0.0061 (11)	−0.0272 (12)
C18	0.0564 (15)	0.0518 (17)	0.0673 (19)	−0.0064 (13)	−0.0096 (14)	−0.0413 (15)
C19	0.0628 (16)	0.0637 (19)	0.0514 (16)	0.0021 (14)	−0.0078 (13)	−0.0426 (15)
C20	0.0448 (12)	0.0485 (15)	0.0347 (12)	0.0034 (11)	−0.0042 (10)	−0.0229 (11)
C21	0.0577 (15)	0.0673 (19)	0.0321 (13)	0.0035 (14)	0.0048 (11)	−0.0235 (13)
C22	0.0520 (15)	0.0549 (17)	0.0351 (13)	−0.0078 (13)	0.0126 (11)	−0.0108 (12)
C23	0.0424 (12)	0.0421 (14)	0.0358 (13)	−0.0077 (11)	0.0062 (10)	−0.0119 (11)
C24	0.0324 (10)	0.0346 (12)	0.0303 (11)	0.0019 (9)	−0.0046 (9)	−0.0156 (9)
O1	0.171 (8)	0.152 (8)	0.109 (6)	−0.028 (6)	0.017 (5)	−0.050 (5)
C25	0.202 (9)	0.147 (8)	0.135 (8)	−0.061 (7)	0.015 (7)	−0.069 (7)
C26	0.142 (7)	0.100 (6)	0.083 (5)	−0.003 (5)	−0.017 (5)	−0.049 (5)
C27	0.172 (8)	0.157 (8)	0.094 (6)	−0.049 (6)	0.027 (6)	−0.054 (6)
O1'	0.147 (7)	0.097 (6)	0.107 (7)	−0.089 (5)	0.063 (6)	−0.030 (4)
C25'	0.139 (8)	0.088 (6)	0.095 (7)	−0.033 (6)	0.029 (6)	−0.058 (5)
C26'	0.144 (8)	0.169 (10)	0.094 (7)	−0.052 (7)	0.020 (6)	−0.046 (7)
C27'	0.149 (9)	0.152 (10)	0.203 (12)	−0.045 (8)	0.008 (8)	−0.059 (8)

### Geometric parameters (Å, °)

**Table:**

Zn1—N1	2.190 (2)	C10—C11	1.399 (3)
Zn1—N2	2.198 (2)	C10—H10	0.9300
Zn1—N3	2.157 (2)	C11—H11	0.9300
Zn1—N4	2.275 (2)	C13—C17	1.407 (3)
Zn1—O1w	2.090 (2)	C13—C24	1.438 (3)
Zn1—Cl1	2.3520 (6)	C14—C15	1.391 (4)
O1W—H1W1	0.85 (3)	C14—H14	0.9300
O1W—H1W2	0.84 (3)	C15—C16	1.369 (4)
N1—C2	1.319 (3)	C15—H15	0.9300
N1—C1	1.360 (3)	C16—C17	1.400 (4)
N2—C11	1.322 (3)	C16—H16	0.9300
N2—C12	1.350 (3)	C17—C18	1.431 (4)
N3—C14	1.329 (3)	C18—C19	1.349 (4)
N3—C13	1.355 (3)	C18—H18	0.9300
N4—C23	1.314 (3)	C19—C20	1.423 (4)
N4—C24	1.365 (3)	C19—H19	0.9300
N5—C25'	1.320 (10)	C20—C24	1.404 (3)
N5—C25	1.325 (11)	C20—C21	1.416 (4)
N5—C26'	1.424 (8)	C21—C22	1.354 (4)
N5—C26	1.431 (7)	C21—H21	0.9300
N5—C27'	1.432 (9)	C22—C23	1.397 (4)
N5—C27	1.487 (7)	C22—H22	0.9300
C1—C5	1.410 (3)	C23—H23	0.9300
C1—C12	1.435 (3)	O1—C25	1.242 (9)
C2—C3	1.397 (3)	C25—H25	0.9300
C2—H2	0.9300	C26—H26A	0.9600
C3—C4	1.357 (4)	C26—H26B	0.9600
C3—H3	0.9300	C26—H26C	0.9600
C4—C5	1.401 (4)	C27—H27A	0.9600
C4—H4	0.9300	C27—H27B	0.9600
C5—C6	1.433 (4)	C27—H27C	0.9600
C6—C7	1.332 (4)	O1'—C25'	1.291 (19)
C6—H6	0.9300	C25'—H25'	0.9300
C7—C8	1.428 (4)	C26'—H26D	0.9600
C7—H7	0.9300	C26'—H26E	0.9600
C8—C9	1.408 (4)	C26'—H26F	0.9600
C8—C12	1.414 (3)	C27'—H27D	0.9600
C9—C10	1.357 (4)	C27'—H27E	0.9600
C9—H9	0.9300	C27'—H27F	0.9600
			
O1W—Zn1—N3	96.59 (7)	N2—C11—C10	122.9 (2)
O1W—Zn1—N1	167.57 (6)	N2—C11—H11	118.5
N3—Zn1—N1	91.38 (7)	C10—C11—H11	118.5
O1W—Zn1—N2	93.77 (7)	N2—C12—C8	123.0 (2)
N3—Zn1—N2	160.59 (7)	N2—C12—C1	118.07 (19)
N1—Zn1—N2	75.98 (7)	C8—C12—C1	118.9 (2)
O1W—Zn1—N4	86.13 (7)	N3—C13—C17	122.6 (2)
N3—Zn1—N4	75.15 (6)	N3—C13—C24	117.74 (19)
N1—Zn1—N4	86.72 (6)	C17—C13—C24	119.6 (2)
N2—Zn1—N4	89.27 (7)	N3—C14—C15	123.4 (2)
O1W—Zn1—Cl1	93.08 (5)	N3—C14—H14	118.3
N3—Zn1—Cl1	97.30 (5)	C15—C14—H14	118.3
N1—Zn1—Cl1	95.36 (5)	C16—C15—C14	119.0 (2)
N2—Zn1—Cl1	98.48 (5)	C16—C15—H15	120.5
N4—Zn1—Cl1	172.25 (5)	C14—C15—H15	120.5
Zn1—O1W—H1W1	114 (2)	C15—C16—C17	119.6 (2)
Zn1—O1W—H1W2	120 (2)	C15—C16—H16	120.2
H1W1—O1W—H1W2	110 (3)	C17—C16—H16	120.2
C2—N1—C1	118.2 (2)	C16—C17—C13	117.6 (2)
C2—N1—Zn1	127.51 (15)	C16—C17—C18	123.1 (2)
C1—N1—Zn1	114.19 (15)	C13—C17—C18	119.3 (2)
C11—N2—C12	118.1 (2)	C19—C18—C17	120.6 (2)
C11—N2—Zn1	127.78 (16)	C19—C18—H18	119.7
C12—N2—Zn1	114.09 (15)	C17—C18—H18	119.7
C14—N3—C13	117.80 (19)	C18—C19—C20	121.6 (2)
C14—N3—Zn1	125.44 (16)	C18—C19—H19	119.2
C13—N3—Zn1	116.76 (14)	C20—C19—H19	119.2
C23—N4—C24	117.9 (2)	C24—C20—C21	116.6 (2)
C23—N4—Zn1	129.65 (17)	C24—C20—C19	119.4 (2)
C24—N4—Zn1	112.42 (13)	C21—C20—C19	124.0 (2)
C25'—N5—C26'	115.4 (8)	C22—C21—C20	120.2 (2)
C25—N5—C26	128.7 (7)	C22—C21—H21	119.9
C25'—N5—C27'	119.3 (10)	C20—C21—H21	119.9
C26'—N5—C27'	125.4 (9)	C21—C22—C23	118.9 (2)
C25—N5—C27	119.5 (8)	C21—C22—H22	120.6
C26—N5—C27	111.8 (6)	C23—C22—H22	120.6
N1—C1—C5	122.3 (2)	N4—C23—C22	123.6 (2)
N1—C1—C12	117.64 (19)	N4—C23—H23	118.2
C5—C1—C12	120.0 (2)	C22—C23—H23	118.2
N1—C2—C3	122.9 (2)	N4—C24—C20	122.8 (2)
N1—C2—H2	118.6	N4—C24—C13	117.76 (19)
C3—C2—H2	118.6	C20—C24—C13	119.4 (2)
C4—C3—C2	119.5 (3)	O1—C25—N5	118.4 (13)
C4—C3—H3	120.2	O1—C25—H25	120.8
C2—C3—H3	120.2	N5—C25—H25	120.8
C3—C4—C5	119.6 (2)	N5—C26—H26A	109.5
C3—C4—H4	120.2	N5—C26—H26B	109.5
C5—C4—H4	120.2	N5—C26—H26C	109.5
C4—C5—C1	117.3 (2)	N5—C27—H27A	109.5
C4—C5—C6	123.9 (2)	N5—C27—H27B	109.5
C1—C5—C6	118.8 (2)	N5—C27—H27C	109.5
C7—C6—C5	121.4 (2)	O1'—C25'—N5	122.1 (11)
C7—C6—H6	119.3	O1'—C25'—H25'	118.9
C5—C6—H6	119.3	N5—C25'—H25'	118.9
C6—C7—C8	121.4 (2)	N5—C26'—H26D	109.5
C6—C7—H7	119.3	N5—C26'—H26E	109.5
C8—C7—H7	119.3	H26D—C26'—H26E	109.5
C9—C8—C12	116.8 (2)	N5—C26'—H26F	109.5
C9—C8—C7	123.9 (2)	H26D—C26'—H26F	109.5
C12—C8—C7	119.4 (3)	H26E—C26'—H26F	109.5
C10—C9—C8	119.7 (2)	N5—C27'—H27D	109.5
C10—C9—H9	120.1	N5—C27'—H27E	109.5
C8—C9—H9	120.1	H27D—C27'—H27E	109.5
C9—C10—C11	119.4 (3)	N5—C27'—H27F	109.5
C9—C10—H10	120.3	H27D—C27'—H27F	109.5
C11—C10—H10	120.3	H27E—C27'—H27F	109.5
			
O1W—Zn1—N1—C2	−146.2 (3)	C7—C8—C9—C10	−178.8 (3)
N3—Zn1—N1—C2	−16.20 (19)	C8—C9—C10—C11	−1.1 (4)
N2—Zn1—N1—C2	178.7 (2)	C12—N2—C11—C10	0.0 (4)
N4—Zn1—N1—C2	−91.24 (19)	Zn1—N2—C11—C10	178.9 (2)
Cl1—Zn1—N1—C2	81.27 (19)	C9—C10—C11—N2	0.6 (4)
O1W—Zn1—N1—C1	36.5 (4)	C11—N2—C12—C8	−0.2 (3)
N3—Zn1—N1—C1	166.52 (14)	Zn1—N2—C12—C8	−179.21 (17)
N2—Zn1—N1—C1	1.41 (14)	C11—N2—C12—C1	179.3 (2)
N4—Zn1—N1—C1	91.48 (14)	Zn1—N2—C12—C1	0.3 (2)
Cl1—Zn1—N1—C1	−96.01 (14)	C9—C8—C12—N2	−0.2 (3)
O1W—Zn1—N2—C11	7.4 (2)	C7—C8—C12—N2	179.5 (2)
N3—Zn1—N2—C11	129.6 (2)	C9—C8—C12—C1	−179.8 (2)
N1—Zn1—N2—C11	−179.8 (2)	C7—C8—C12—C1	0.0 (3)
N4—Zn1—N2—C11	93.4 (2)	N1—C1—C12—N2	1.0 (3)
Cl1—Zn1—N2—C11	−86.3 (2)	C5—C1—C12—N2	−177.85 (19)
O1W—Zn1—N2—C12	−173.77 (15)	N1—C1—C12—C8	−179.48 (19)
N3—Zn1—N2—C12	−51.5 (3)	C5—C1—C12—C8	1.7 (3)
N1—Zn1—N2—C12	−0.90 (15)	C14—N3—C13—C17	2.6 (3)
N4—Zn1—N2—C12	−87.70 (15)	Zn1—N3—C13—C17	−177.17 (17)
Cl1—Zn1—N2—C12	92.54 (15)	C14—N3—C13—C24	−176.9 (2)
O1W—Zn1—N3—C14	−99.15 (19)	Zn1—N3—C13—C24	3.3 (3)
N1—Zn1—N3—C14	90.41 (19)	C13—N3—C14—C15	−2.3 (4)
N2—Zn1—N3—C14	139.0 (2)	Zn1—N3—C14—C15	177.5 (2)
N4—Zn1—N3—C14	176.6 (2)	N3—C14—C15—C16	0.2 (4)
Cl1—Zn1—N3—C14	−5.17 (19)	C14—C15—C16—C17	1.7 (4)
O1W—Zn1—N3—C13	80.64 (16)	C15—C16—C17—C13	−1.3 (4)
N1—Zn1—N3—C13	−89.80 (16)	C15—C16—C17—C18	178.0 (3)
N2—Zn1—N3—C13	−41.2 (3)	N3—C13—C17—C16	−0.9 (4)
N4—Zn1—N3—C13	−3.56 (15)	C24—C13—C17—C16	178.7 (2)
Cl1—Zn1—N3—C13	174.62 (15)	N3—C13—C17—C18	179.8 (2)
O1W—Zn1—N4—C23	82.8 (2)	C24—C13—C17—C18	−0.6 (3)
N3—Zn1—N4—C23	−179.3 (2)	C16—C17—C18—C19	−178.5 (3)
N1—Zn1—N4—C23	−87.0 (2)	C13—C17—C18—C19	0.8 (4)
N2—Zn1—N4—C23	−11.0 (2)	C17—C18—C19—C20	−0.2 (4)
O1W—Zn1—N4—C24	−94.44 (15)	C18—C19—C20—C24	−0.6 (4)
N3—Zn1—N4—C24	3.43 (14)	C18—C19—C20—C21	178.9 (3)
N1—Zn1—N4—C24	95.73 (15)	C24—C20—C21—C22	−0.8 (4)
N2—Zn1—N4—C24	171.73 (15)	C19—C20—C21—C22	179.7 (3)
C2—N1—C1—C5	−0.5 (3)	C20—C21—C22—C23	0.4 (4)
Zn1—N1—C1—C5	177.05 (16)	C24—N4—C23—C22	−0.4 (4)
C2—N1—C1—C12	−179.31 (19)	Zn1—N4—C23—C22	−177.58 (18)
Zn1—N1—C1—C12	−1.8 (2)	C21—C22—C23—N4	0.3 (4)
C1—N1—C2—C3	1.0 (3)	C23—N4—C24—C20	0.0 (3)
Zn1—N1—C2—C3	−176.18 (17)	Zn1—N4—C24—C20	177.58 (17)
N1—C2—C3—C4	−0.4 (4)	C23—N4—C24—C13	179.4 (2)
C2—C3—C4—C5	−0.8 (4)	Zn1—N4—C24—C13	−3.0 (2)
C3—C4—C5—C1	1.2 (3)	C21—C20—C24—N4	0.7 (3)
C3—C4—C5—C6	−179.4 (2)	C19—C20—C24—N4	−179.8 (2)
N1—C1—C5—C4	−0.6 (3)	C21—C20—C24—C13	−178.7 (2)
C12—C1—C5—C4	178.2 (2)	C19—C20—C24—C13	0.8 (3)
N1—C1—C5—C6	−180.0 (2)	N3—C13—C24—N4	0.0 (3)
C12—C1—C5—C6	−1.2 (3)	C17—C13—C24—N4	−179.6 (2)
C4—C5—C6—C7	179.6 (2)	N3—C13—C24—C20	179.4 (2)
C1—C5—C6—C7	−1.1 (4)	C17—C13—C24—C20	−0.1 (3)
C5—C6—C7—C8	2.9 (4)	C26—N5—C25—O1	0.8 (4)
C6—C7—C8—C9	177.4 (3)	C27—N5—C25—O1	179.3 (3)
C6—C7—C8—C12	−2.3 (4)	C26'—N5—C25'—O1'	0.8 (3)
C12—C8—C9—C10	0.9 (4)	C27'—N5—C25'—O1'	−179.9 (3)

### Hydrogen-bond geometry (Å, °)

**Table:**

*D*—H···*A*	*D*—H	H···*A*	*D*···*A*	*D*—H···*A*
O1w—H1w1···Cl2	0.85 (3)	2.29 (3)	3.112 (2)	163 (3)
O1w—H1w2···Cl2^i^	0.84 (3)	2.24 (3)	3.079 (2)	172 (3)

Symmetry codes: (i) −*x*+1, −*y*+2, −*z*+1.
